# Multimodal predictors of response to intravenous ketamine in treatment-resistant depression: toward personalized treatment in major depressive and bipolar disorders

**DOI:** 10.1017/S1092852926101187

**Published:** 2026-09-28

**Authors:** Magda Malewska-Kasprzak, Filip Rybakowski, Monika Dmitrzak-Weglarz

**Affiliations:** 1 Adult Psychiatry, Poznan University of Medical Sciences, Poland; 2 Department of Psychiatric Genetics, Poznan University of Medical Sciences, Poznan, Poland

**Keywords:** Intravenous ketamine, treatment-resistant depression, major depressive disorder, bipolar disorder, CNS biomarkers, neurophysiology, personalized psychiatry

## Abstract

Intravenous ketamine is a rapid-acting intervention for treatment-resistant depression (TRD) in adults with major depressive disorder and bipolar disorder (BD). While efficacy is established, a comprehensive integration of clinical, neurophysiological, biomarker, metabolic, and cognitive predictors remains lacking, limiting precision CNS-targeted therapy. To synthesize recent evidence on multimodal predictors of intravenous ketamine response in TRD and propose a preliminary clinical framework to guide personalized treatment. Following PRISMA guidelines, Scopus, PubMed, EMBASE, and MEDLINE were searched for English-language, peer-reviewed studies published within the last 5 years. Eligible studies included adults receiving intravenous ketamine for depressive disorders. Data were descriptively synthesized to identify consistent predictors, evidence clusters, and research gaps. The review included 150 studies. Key predictors of ketamine response included baseline depressive severity, comorbid anxiety, atypical depression and sleep phenotypes, inflammatory and neuroplastic biomarkers (eg, monocyte count, BDNF), metabolic factors (BMI, vitamin B12), genetic/epigenetic variants, neurophysiological signatures (EEG, TMS, fMRI connectivity), cognitive performance, and early symptomatic improvement. Integrative analysis suggests that glutamatergic, circadian, and inflammatory mechanisms interact to shape interindividual variability. Limitations include methodological heterogeneity and scarce long-term outcome data. Intravenous ketamine response in TRD is multifactorial, reflecting clinical phenotype, CNS biomarkers, neurophysiology, metabolism, and genetics. This review proposes a preliminary research framework and pilot clinical questionnaire intended to facilitate future investigation of individualized treatment approaches. However, most proposed predictors remain exploratory and require prospective validation before clinical implementation.

## Introduction

Treatment-resistant depression (TRD) affects a substantial proportion of patients with major depressive disorder (MDD) and bipolar disorder (BD), leading to chronic functional impairment, elevated suicide risk, and significant social and economic burden (Alexander et al., 2023). Defined as the failure to respond to at least two adequate antidepressant trials, TRD is associated with higher relapse rates, cognitive deficits, and suicidal behaviors.^1^ ^,^^2^ Both unipolar and bipolar depressive episodes can manifest as TRD, highlighting the need for rapid and effective interventions.

The discovery of intravenous ketamine as a rapid-acting antidepressant has catalyzed extensive research into its mechanisms of action, efficacy, and predictors of response. Over the past 5 years, multiple randomized controlled trials, neuroimaging studies, genetic analyses, and observational studies have begun to identify clinical, neurophysiological, metabolic, and biomarker factors that influence therapeutic outcomes (Aslam et al., 2025). Despite this progress, a comprehensive framework integrating these multimodal predictors remains lacking, limiting precision CNS-targeted approaches in clinical practice.

The objective of this review is to synthesize the latest evidence on predictors of intravenous ketamine response in TRD and propose an integrative clinical framework to guide personalized treatment. Unlike prior reviews that focus predominantly on efficacy, this work incorporates clinical phenotypes, sleep and metabolic profiles, inflammatory and neuroplastic biomarkers, genetic/epigenetic factors, and neurophysiological signatures, with the aim of identifying candidate predictors for future individualized treatment strategies. At present, no biomarker has sufficient evidence to support routine clinical decision-making in ketamine treatment.

## Review methodology

### Strategy for literature search

This review was conducted as a structured narrative review informed by PRISMA principles for literature identification and reporting.

In the first stage, we identified online repositories of biomedical literature, including Scopus, EMBASE, PubMed, and MEDLINE. Articles were retrieved from these databases using the following search strategy: ([ketamine] AND ((intravenous) OR (IV) OR (infusion)) AND [depression]). Duplicate records were automatically removed using the ZOTERO reference manager (PMID: 37684721).

Further screening of the retrieved records was performed according to predefined inclusion and exclusion criteria.

**Inclusion criteria:**

- Full-text articles;
- Published in peer-reviewed journals;
- Published within the last 5 years;
- Published in English;
- Human studies;
- Adults aged 19–65 years.

**Exclusion criteria:**

- Duplicate records;
- Diagnoses other than depression;
- Non-human studies;
- Lack of full-text availability in English;
- Non-IV ketamine administration;
- Combined treatments;
- Case reports;
- Studies involving children or adolescents;
- Other non-relevant topics.

Additionally, a manual search was conducted to identify specific biomarkers mentioned in the text, yielding *x* additional publications.

**Figure 1.**
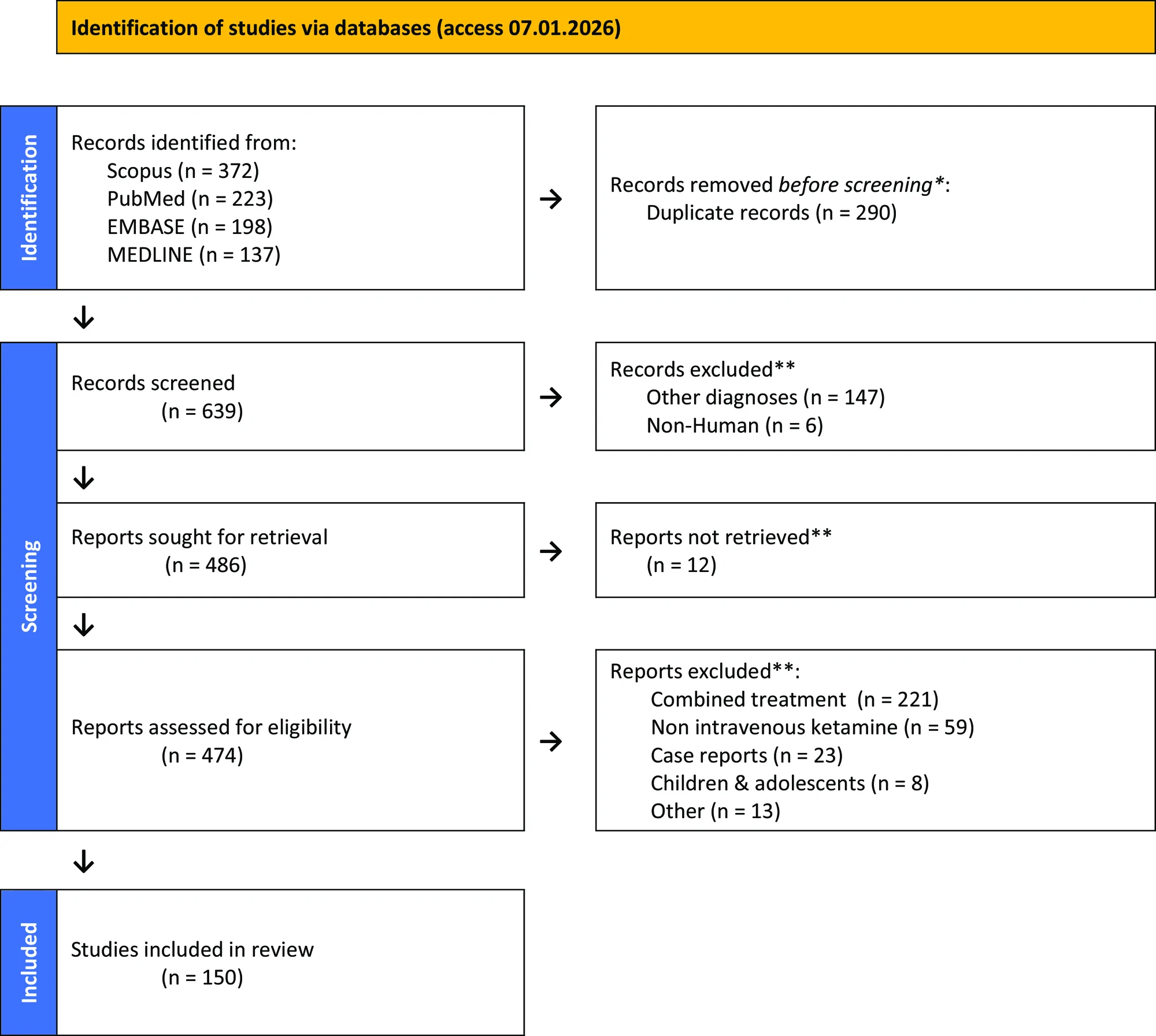
Flow diagram of a systematic selection of intravenous ketamine publications according to PRISMA guidelines (See also section “Strategy for literature search”). *Record excluded using automated tools built-in database and bibliography manager. **Record excluded by a researcher. Figure 1. long description.

Source: Page MJ, McKenzie JE, Bossuyt PM, Boutron I, Hoffmann TC, Mulrow CD, et al. The PRISMA 2020 statement: an updated guideline for reporting systematic reviews. BMJ 2021;372:n71. doi:10.1136/bmj.n71 (http://www.prisma-statement.org/).

Publications meeting all the above criteria underwent a full-text review. Figure 1 presents a schematic representation of the publication selection process.

## Mechanism of action of intravenous ketamine

Intravenous ketamine is a non-competitive NMDA receptor antagonist acting primarily on GABAergic interneurons in the prefrontal cortex and hippocampus, leading to disinhibition of glutamatergic neurons, increased glutamate release, and AMPA receptor activation.^3^ ^–^^5^ This triggers mTORC1 signaling and BDNF upregulation, promoting rapid synaptic remodeling and neuroplasticity in mood-regulating circuits, which underlies ketamine’s fast antidepressant effects.^2^ ^,^^6^ ^,^^7^

Modulation of lateral habenula (LHb) activity is another key mechanism. Hyperactive LHb in depression suppresses midbrain monoaminergic nuclei via NMDA-dependent burst firing; ketamine blocks these discharges, restoring dopaminergic and serotonergic balance and contributing to rapid clinical improvement.^8^ ^–^^10^ Resting-state fMRI shows that ketamine alters LHb connectivity with cortical regions involved in cognitive control and emotion regulation, suggesting network-level effects beyond synaptic mechanisms.^11^ ^,^^12^

The opioid system also contributes: both acute and sustained antidepressant effects can be blocked by opioid antagonists, independent of psychotomimetic effects.^4^ ^,^^13^ Additionally, ketamine has anti-inflammatory and neuroprotective properties, reducing proinflammatory cytokines and oxidative stress markers, relevant in inflammatory subtypes of depression.^4^ ^,^^14^

## Efficacy of intravenous ketamine in MDD and BD

In most patients, the effect persists for 3–7 days, supporting the use of repeated infusion series or maintenance treatment strategies.^1^ ^,^^2^ ^,^^15^ Numerous clinical studies have shown that early symptom improvement after the first or second infusion is a significant predictor of treatment response.^16^ Data from the Canadian Rapid Treatment of Depression Centre indicate that patients achieving ≥20% reduction in QIDS-SR16 scores after 1–2 infusions experienced significantly greater improvement by the end of a four-infusion protocol.^17^ At the same time, some patients without an early response also showed clinically meaningful symptom reduction after the full treatment course, highlighting the importance of completing the planned series of infusions before assessing treatment nonresponse.^18^ The number of infusions, intervals between them, and maintenance strategies are clinically relevant but do not eliminate the risk of relapse.^19^ ^–^^22^ ^,^^23^

In BD, ketamine exhibits comparable efficacy in treating depressive episodes without significantly increasing the risk of manic switching when mood stabilizers are maintained. Its effects are particularly important in patients with severe anhedonia and suicidal ideation, where rapid therapeutic action is clinically critical.^1^ ^,^^2^ ^,^^24^

## Biomarkers and predictors of response to intravenous ketamine in treatment-resistant depression

Therapeutic response to intravenous ketamine in TRD is influenced by interactions among inflammatory processes, neuroplasticity, and genetic factors.^25^ ^,^^26^ ^,^^27^ Identifying these biomarkers may contribute to future precision psychiatry approaches; however, currently available evidence is insufficient to support routine patient selection based on biomarker profiles.^28^

### Inflammatory biomarkers

Elevated proinflammatory cytokines, including IL-6, TNF-α, and CRP, are associated with poor response to conventional antidepressants, particularly serotonergic and noradrenergic agents.^28^ Ketamine’s immunomodulatory effects, such as reduced microglial activation and modulation of cytokine signaling, may enhance efficacy in patients with elevated inflammation.^29^ ^,^^30^ Recent evidence highlights baseline absolute monocyte count (AMC) as a stronger predictor of ketamine response than CRP, correlating with symptom improvement independent of age, sex, or BMI.^31^ Nevertheless, these findings originate from a limited number of studies and have not yet been independently replicated in large prospective cohorts.

### Neuroplasticity biomarkers

BDNF is among the most frequently investigated candidate biomarkers of ketamine response and a pharmacodynamic marker of ketamine response; higher pre-treatment levels or post-infusion increases are linked to better clinical outcomes.^32^ ^,^^33^ However, results remain inconsistent across studies, and no consensus currently supports its clinical use as a predictive marker. Other neuroplasticity-related markers, including VEGF, neurofilament light chain, complement components, and selected cytokines, also correlate with clinical response, reflecting synaptic remodeling and CNS reparative processes.^34^ ^–^^38^

### Genetic and epigenetic predictors

Genetic variants modulate ketamine efficacy and durability. BDNF Val66Met polymorphism affects secretion and response dynamics; CYP2B6 variants influence pharmacokinetics; GRIN2B and other NMDA receptor gene variants are linked to efficacy and tolerability.^19^ ^,^^40^ ^–^^43^ GWAS suggest polygenic influences, though clinical application remains limited.^41^ ^,^^43^ Epigenetic modifications, including DNA methylation and microRNA regulation of the HPA axis, neuroplasticity, and immune genes, further modulate ketamine sensitivity,^28^ ^,^^44^ ^,^^45^ (Table 1). Despite growing interest in genetic and epigenetic predictors, available findings are characterized by small sample sizes, inconsistent replication, and limited predictive performance. Consequently, genetic testing is not currently recommended for routine clinical decisions.

**Table 1.** Biomarkers to Intravenous Ketamine in TRD Table 1. long description.

Biomarker type	Examples	Association with ketamine response	Evidence level	Clinical readiness
Inflammatory	IL-6, TNF-α, AMC	Higher AMC is associated with a better antidepressant response, whereas CRP has not shown consistent predictive value.	Prospective observational studies^31^ ^,^^29^ ^,^^30^	Preliminary evidence
Neuroplasticity	BDNF, VEGF, NfL, complement system components	Higher baseline or post-infusion BDNF is associated with better and more sustained response; changes in VEGF and NfL may reflect ketamine-induced synaptic plasticity.	RCTs and observational studies^32^ ^,^^38^ ^,^^39^ ^,^^35^	Preliminary evidence
Genetic/Epigenetic	BDNF Val66Met, CYP2B6, GRIN2B	Genetic variants may influence treatment response, durability, and tolerability, while epigenetic modifications (DNA methylation, microRNA expression) may affect treatment sensitivity.	GWAS and candidate gene studies^40^ ^,^^28^ (Rodrigues et al., 2024)	Research use only

## Clinical, metabolic, and neurophysiological factors influencing ketamine efficacy

Efficacy of intravenous ketamine in TRD depends on a complex interaction of clinical, metabolic, familial, and neurophysiological factors, which is crucial for therapy personalization.^46^

### Characteristics of depression

The clinical characteristics of a depressive episode represent one of the most well-documented and consistent predictors of response to intravenous ketamine. Numerous studies have demonstrated that specific symptom domains—particularly the severity of anhedonia, presence of suicidal ideation, and comorbid anxiety symptoms—significantly influence both the likelihood and temporal dynamics of response.^47^ Ketamine exhibits particularly rapid and robust anti-anhedonic and anti-suicidal effects, which clearly differentiate it from conventional antidepressants with delayed onset and limited efficacy in these domains.^48^ ^,^^49^

The depressive symptom profile qualitatively modulates treatment efficacy. Patients with non-melancholic, atypical, or anxiety-predominant depression may show a different, often faster response to ketamine than those with classical melancholic depression.^50^ These differences likely reflect distinct neurobiological mechanisms, including the degree of stress axis involvement, glutamatergic system engagement, and networks regulating emotion and reward.^51^ At the same time, severe anxiety symptoms have in some studies been associated with slower or less durable responses, highlighting population heterogeneity and potential moderating factors such as autonomic arousal or comorbid anxiety disorders.^38^ ^,^^43^ ^,^^52^ ^–^^54^

The presence of psychotic features has received relatively limited investigation. Although psychotic depression is not considered an absolute contraindication to intravenous ketamine administered under appropriate psychiatric supervision, available evidence is insufficient to determine whether psychotic symptoms independently influence treatment response. Additional prospective studies are warranted.^55^

In BD, disease course has particular prognostic significance, including history of manic or mixed episodes and current mood stabilization. Ketamine can be effective in treating depressive episodes in BD, but its use carries the risk of mood destabilization, including induction of hypomanic or manic symptoms. Clinical data indicate that patients with Bipolar I disorder, particularly those with a history of rapid cycling or previous manic episodes, should remain on mood stabilizers before initiation of intravenous ketamine treatment in order to minimize the risk of affective switching. This recommendation is primarily based on expert consensus and clinical practice guidelines rather than robust randomized controlled trial evidence.^37^ ^,^^48^ ^,^^56^ ^,^^57^

### Sleep disturbances

They are common in TRD; up to 94% report significant sleep problems, and over 60% present more than one sleep phenotype, including hypersomnia, early insomnia, and REM/SWS architecture changes.^57^ ^,^^58^ Clinical data indicate that patients with hypersomnia and atypical depression features show greater and faster symptom reduction after ketamine compared to those with melancholic depression, suggesting a link between neurovegetative depression profile and glutamatergic.^38^ ^,^^43^ ^,^^52^ ^–^^54^ ^,^^59^ Mechanistically, ketamine increases glutamate release in the prefrontal cortex and may enhance neuroplasticity in areas regulating sleep and mood, potentially explaining higher efficacy in certain depression phenotypes.^2^ ^,^^7^ Objective sleep measures further support these clinical observations. Several polysomnographic studies have demonstrated that ketamine administration increases slow-wave sleep (SWS), enhances slow-wave activity, and modulates REM sleep architecture. These changes correlate with synaptic plasticity and antidepressant response.^60^ Because patients with atypical depression and hypersomnia often exhibit disturbances in SWS regulation, these findings provide a plausible neurobiological explanation for their greater responsiveness to intravenous ketamine. Nevertheless, the number of polysomnographic studies remains limited, and larger prospective investigations are required before PSG markers can be considered clinically useful predictors of treatment outcome.^61^

### Degree of treatment resistance

Degree of TRD is another strong predictor.^38^ ^,^^43^ ^,^^52^ ^–^^54^ Patients with less advanced treatment resistance or fewer previously failed evidence-based treatments generally achieve better outcomes than those with more advanced treatment resistance, prolonged psychiatric hospitalization, or multiple previous treatment failures.^62^ ^,^^63^ Previous treatment failures, including electroconvulsive therapy (ECT), may reflect a greater degree of illness refractoriness. However, current evidence remains insufficient to conclude that prior ECT nonresponse independently predicts reduced response to intravenous ketamine. Importantly, available studies rarely distinguish between lifetime exposure to ECT and failure of ECT during the current depressive episode, limiting interpretation of its prognostic value. Therefore, previous ECT nonresponse should not currently be considered an independent contraindication to intravenous ketamine treatment, although further prospective studies are needed.^64^ ^,^^65^ Comorbid anxiety symptoms may increase the likelihood of favorable response, although findings are inconsistent and may depend on methodology and study population.^46^ ^,^^49^ In BD, disease course has particular prognostic significance, including history of manic or mixed episodes and current mood stabilization. Ketamine can be effective in treating depressive episodes in BD, but its use carries the risk of mood destabilization, including induction of hypomanic or manic symptoms.^66^ Clinical data indicate that adequate pharmacological stabilization prior to ketamine administration is a key safety factor and may also influence treatment efficacy.^37^ ^,^^48^ ^,^^56^ ^,^^57^ ^,^^67^

### Family history and substance use

One well-documented predictor is a positive first-degree family history of alcohol use disorder (AUD).^68^ Studies showed that TRD patients with positive family AUD history responded more strongly and rapidly to ketamine within hours.^68^ ^,^^69^ Subsequent meta-analyses confirmed that positive AUD family history is associated with significantly better response at both 1 and 7 days post-infusion.^14^ Personal substance use history, especially alcohol, may also influence ketamine efficacy, though data are mixed. Some studies suggest greater response in those with prior AUD, while others show no significant difference, potentially related to abstinence duration, addiction severity, or psychosocial factors.^68^ ^,^^70^ Childhood trauma, higher anhedonia severity, and lack of melancholic features were also associated with better response in some studies, though contradictory findings exist.^46^ ^,^^52^ ^,^^71^

### Metabolic parameters

Metabolic parameters, including body mass index (BMI) and vitamin B12 levels, may modulate response to ketamine, although data remain partially inconsistent.^27^ In some studies, higher BMI was associated with better therapeutic response, potentially due to effects on ketamine pharmacokinetics or low-grade inflammation, while other analyses did not confirm this relationship.^27^ ^,^^72^ ^–^^74^These discrepancies may result from differences in dosing regimens, response assessment methods, and heterogeneity of study populations.^72^

Vitamin B12 plays a critical role in central nervous system function, supporting DNA methylation, neurotransmitter synthesis, and myelin integrity.^75^ B12 deficiency has been repeatedly linked to poorer antidepressant response, whereas adequate levels or supplementation may enhance the efficacy of biological therapies, including ketamine.^73^ ^,^^76^ ^,^^77^

### Biological sex, age, and concomitant pharmacotherapy

The influence of biological sex on ketamine efficacy remains inconclusive. Most clinical studies do not report significant differences in therapeutic response between females and males in terms of either magnitude or duration of antidepressant effect. Nonetheless, some reports have suggested slightly better response in males within the first 24 hours post-infusion, possibly related to hormonal, pharmacokinetic, or receptor sensitivity differences.^73^ ^,^^78^

Concomitant use of other psychotropic medications is an important factor modulating ketamine efficacy. Attention has focused particularly on benzodiazepines and opioid receptor antagonists such as naltrexone. Some studies have shown attenuation of ketamine’s antidepressant effect with high-dose benzodiazepines, likely due to their inhibitory influence on synaptic plasticity and prefrontal cortex activation. Similarly, opioid receptor blockade has in some studies been associated with reduced ketamine efficacy, suggesting involvement of the opioid system in its mechanism of action.^79^

However, other studies have not confirmed a significant impact of concomitant pharmacotherapy on clinical response, indicating potential modulation by dose, timing, and individual biological differences.^17^ ^,^^46^ Clinically, these factors should be considered when planning ketamine treatment, although they do not always necessitate modification of existing pharmacotherapy.^80^

Age has been investigated as a potential moderator of ketamine response, although findings remain inconsistent. Most studies suggest comparable antidepressant efficacy across adult age groups, whereas older patients are substantially underrepresented in clinical trials.^67^ Consequently, current evidence does not support age as an independent predictor of response, although pharmacokinetic differences and medical comorbidities should be considered when treating elderly patients.^67^ ^,^^80^

### Neuroimaging and neurophysiological factors

Neuroimaging and neurophysiological markers are increasingly recognized as predictors of intravenous ketamine efficacy in TRD, reflecting susceptibility to rapid antidepressant action and response durability.

fMRI studies show baseline functional connectivity of the prefrontal cortex with the cingulate gyrus, hippocampus, and amygdala predicts ketamine response.^81^ ^–^^83^ ^,^^84^ Disrupted networks in TRD are linked to rumination, emotional dysregulation, and cognitive deficits.^85^ ^,^^86^ Ketamine modulates glutamatergic transmission, normalizing connectivity in default mode, executive, and salience networks.^56^ ^,^^81^ ^,^^87^

EEG and MEG markers, including gamma, theta, and alpha power, as well as interregional synchrony, predict ketamine response, with gamma activity reflecting glutamatergic activation and synaptic plasticity.^88^ ^–^^90^ ^,^^91^ MRS shows prefrontal glutamate/GABA ratios and their modulation by ketamine correlate with clinical improvement.^19^ ^,^^81^ ^,^^87^

Pilot protocols in ECT-nonresponsive patients use biweekly ketamine infusions with TMS-EMG–EEG assessments. Measures like intracortical facilitation (ICF) and short-interval cortical inhibition (SICI) reflect glutamatergic/GABAergic balance and ketamine’s mechanism.^92^ ^–^^97^

Resting-state EEG assessing vigilance and attention stability, eg, phase A1 proportion in cyclic alternating pattern (CAP) analysis, correlates with better and more stable ketamine response.^98^ ^,^^99^ Although neuroimaging and neurophysiological measures provide valuable mechanistic insights, their clinical applicability remains limited by high costs, methodological heterogeneity, lack of standardization, and absence of prospective validation studies.

## Cognitive and psychological factors

Cognitive and psychological domains significantly influence ketamine response in TRD. Evidence indicates that cognitive functions: particularly working memory, attention, and cognitive flexibility, mediate rapid antidepressant effects and reductions in suicidal ideation.^100^ ^,^^101^ Cognitive deficits, common in TRD, are associated with poorer prognosis and weaker response to conventional antidepressants, highlighting their potential as moderators of ketamine efficacy.^102^

Post-infusion improvements in executive function have been observed independently of mood changes, suggesting partial dissociation between cognitive and affective effects.^103^ Baseline performance in working memory and attentional shifting may predict more stable and durable clinical responses, likely via prefrontal cortex activation, normalization of executive network function, and restored balance between default mode and salience networks, reducing rumination and cognitive rigidity.^104^ ^,^^105^

Ketamine’s impact on cognition is particularly relevant to suicidal thinking. Reduced cognitive rigidity and enhanced attentional control may mediate rapid decreases in suicidal ideation, often within hours of infusion, independently of overall mood improvement.^14^ ^,^^55^ ^,^^106^ Importantly, baseline suicidal ideation should not be interpreted as a predictor of poor ketamine response.^63^ On the contrary, ketamine consistently demonstrates rapid anti-suicidal effects that frequently occur independently of overall antidepressant response, making suicidality an important clinical characteristic to consider during patient selection.^106^

The role of dissociative and psychomimetic experiences remains debated. Most studies indicate that their intensity is neither necessary nor sufficient for antidepressant efficacy; however, moderate subjective experiences—such as depersonalization, derealization, or perceptual changes—have been correlated with better short-term response.^100^ ^,^^108^ ^,^^109^ These transient experiences may “loosen” entrenched cognitive and emotional patterns, creating a therapeutic window for psychotherapeutic interventions, while excessive or unpleasant experiences can reduce tolerability without improving long-term outcomes,^97^ ^,^^110^ ^,^^111^ (Table 2).

**Table 2.** Clinical, Metabolic, Neurophysiological, and Psychological Predictors of IV Ketamine Response in TRD for MDD and BD Table 2. long description.

Predictor type	Examples/Features	Association with ketamine response	Evidence Level/References	Clinical readiness
Treatment resistance	Number of failed antidepressant trials, prior ECT/rTMS	Less advanced treatment resistance (fewer failed antidepressant trials) → better response; more advanced treatment resistance (multiple failed treatments and hospitalizations) → poorer response.	Observational and clinical studies^62^ ^,^^18^	Preliminary evidence
Sleep phenotype	Hypersomnia, early insomnia, REM/SWS changes	Hypersomnia/atypical sleep → higher response; REM/SWS disturbances → variable.	Clinical studies^58^ ^,^^52^ ^,^^59^	Exploratory evidence
Family/Substance history	Family history of AUD, personal history of substance use	Positive family AUD → faster and stronger response; personal substance use → mixed evidence.	Clinical studies and meta-analyses^68^ ^,^^14^ ^,^^70^	Exploratory evidence
Metabolic factors	Vitamin B12 levels, BMI, childhood trauma	Normal/high B12, higher BMI → better response; childhood trauma → may enhance response.	Observational studies^76^ ^,^^73^ ^,^^107^	Exploratory evidence
Neurophysiological/Neuroimaging	fMRI connectivity (PFC–hippocampus–amygdala), EEG/MEG oscillatory patterns, TMS (SICI/ICF), MRS glutamate/GABA ratio	Baseline network disruption → predicts response; gamma/theta activity and cortical excitation–inhibition balance → strong predictors.	Clinical and pilot studies^56^ ^,^^92^ ^,^^90^ ^,^^89^	Research use only
Cognitive/Psychological	Working memory, attention, cognitive flexibility, dissociative experiences	Better baseline cognitive performance → more stable response; moderate dissociation → may enhance short-term response.	Clinical studies^100^ ^,^^108^ ^,^^109^	Exploratory evidence
Concomitant Medications	Benzodiazepines, opioid antagonists	High doses may attenuate response; minimal doses → better efficacy.	Clinical studies^79^ ^,^^46^	Preliminary evidence

## Potential clinical application and future directions

A multimodal approach integrating inflammatory markers, BDNF levels, genetic polymorphisms, sleep phenotypes, atypical depression features, treatment resistance, comorbid anxiety, early symptom improvement, vitamin B12 level, and substance use history may eventually improve prediction of intravenous ketamine response and supports TRD therapy personalization in MDD and BD.^2^ ^,^^18^

Based on existing studies, several practical clinical recommendations can be formulated to optimize therapy efficacy and minimize adverse events:

### Patient selection

Key to ketamine therapy success is appropriate patient selection, considering response predictors. Several studies suggest that certain patient characteristics may be associated with a higher probability of response, although findings remain inconsistent:

- Atypical depression phenotype, including hypersomnia-type sleep disturbances,^112^ demonstrating higher glutamatergic sensitivity.^113^ ^,^^114^
- Moderate treatment resistance, without a long psychiatric hospitalization history or multiple suicide attempts .^115^ ^,^^116^
- Positive first-degree family history of AUD, correlating with faster and stronger response.^11768^
- Biological factors may indicate greater ketamine sensitivity and serve as predictive indicators.^31^

### Dosing protocols and response monitoring

The most commonly used and well-documented protocol is a 0.5 mg/kg IV infusion over 40 minutes. Clinical practice indicates that repeated infusion series (typically 4–6 infusions over 2–3 weeks) can prolong therapeutic effect and increase response likelihood in TRD patients.^2^ ^,^^7^ ^,^^17^

Early response assessment is critical. After the first or second infusion, there is ≥20% reduction in depression scores (QIDS-SR16), which is a strong predictor of further response.^18^ However, lack of early response does not exclude clinical benefit later; completing the planned protocol before final efficacy assessment is recommended.^118^ ^,^^119^

### Concomitant medications and metabolic factors

Concurrent medications, especially benzodiazepines and opioid antagonists, may modulate ketamine response. High doses may partially attenuate effect; therefore, minimizing use or monitoring influence is recommended.^36^ ^,^^46^ ^,^^79^ ^,^^120^

Metabolic factors, including vitamin B12, BMI, and childhood trauma history, may also influence efficacy. In cases of vitamin B12 deficiency or other adverse metabolic parameters, supplementation or lifestyle modification should be considered to improve treatment response p. 1.^76^

### Integration of biomarkers and neurophysiology

Modern clinical approaches increasingly incorporate biomarkers and neurophysiology in monitoring ketamine response.^121^ TMS combined with EEG and EMG assesses cortical inhibition-excitation balance (SICI/ICF), serving as a promising predictive biomarker.^92^ ^,^^122^ Monitoring biomarkers such as BDNF or inflammatory markers remains investigational and should currently be considered a research tool rather than a standard component of clinical ketamine treatment.^31^ ^,^^112^ ^,^^122^ This type of multimodal approach can significantly support therapy personalization and improve the effectiveness of interventions in TRD.

### Specifics of bipolar disorder

In patients with depression in the course of bipolar affective disorder, it is important to ensure mood stabilization using mood-stabilizing drugs to minimize the risk of switching into a manic or hypomanic episode.^74^ ^,^^123^ Studies indicate that ketamine shows comparable efficacy in depressive episodes of BD and MDD, making it a safe therapeutic option in severe bipolar depression when adequate mood-stabilizing prophylaxis is maintained.^1^ ^,^^2^Based on current data, the following practical clinical implications can be formulated: appropriate patient selection considering depression phenotype, degree of treatment resistance, family history of AUD, and biological markers; use of repeated ketamine infusions with monitoring of early response; control of concomitant medications and metabolic parameters; use of neuroplasticity and neurophysiological biomarkers to predict response; and, in the case of BD, ensuring concurrent mood stabilization.^124^ ^–^^126^ This approach may help optimize treatment outcomes, minimizing the risk of adverse events, and supports the development of personalized psychiatry.^107^ ^,^^127^

Integration of clinical, metabolic, inflammatory, and neuroplastic biomarkers, as well as depression phenotypes, may facilitate personalized treatment approaches. In clinical practice, this means the possibility of:

- selecting patients with a high probability of response,
- adjusting the infusion protocol,
- monitoring early indicators of efficacy,
- minimizing adverse effects,
- developing maintenance treatment strategies and repeated infusions in long-term TRD therapy.^109^

**Figure 2.**
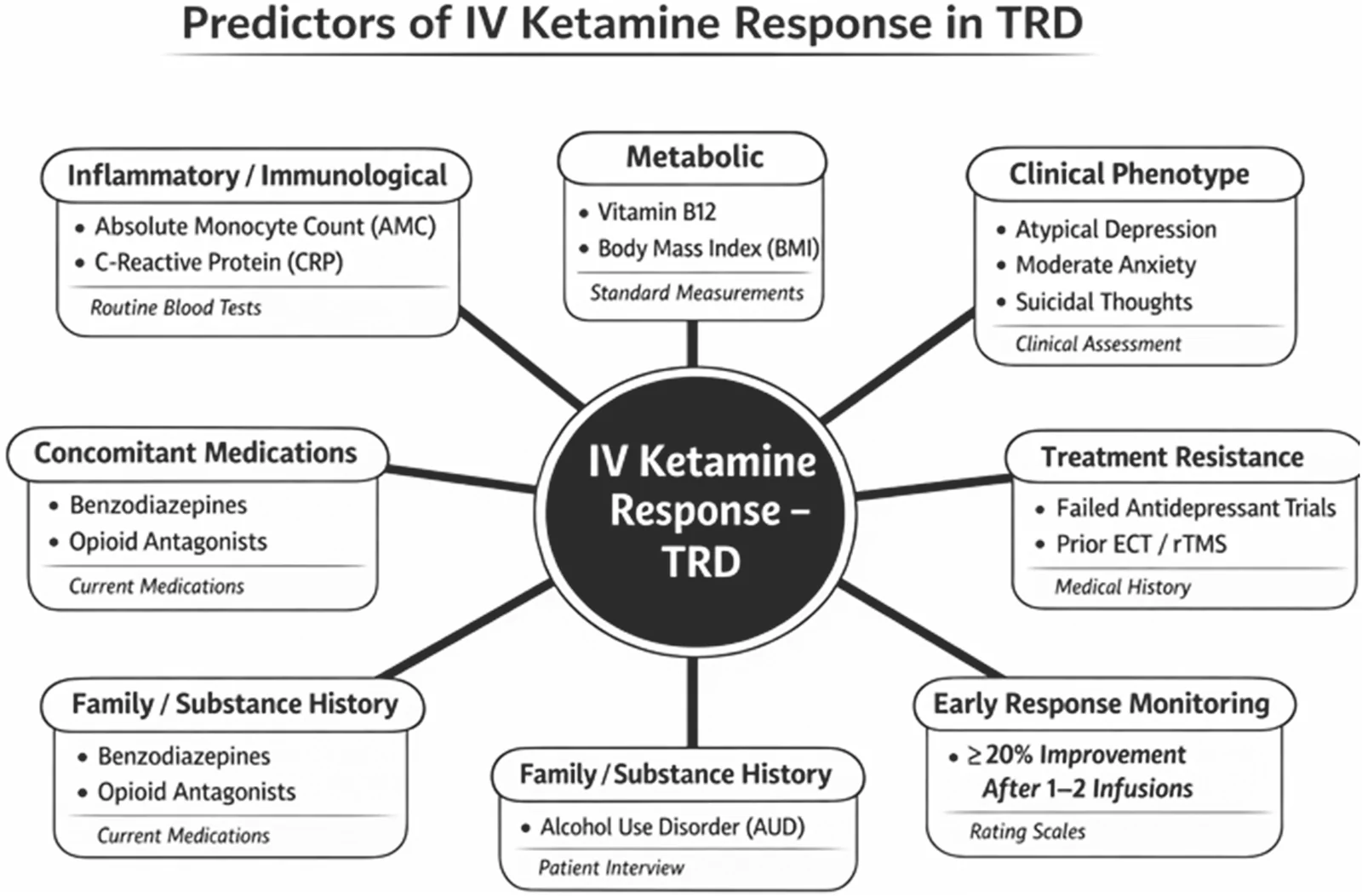
Predictors of IV ketamine response in TRD. Figure 2. long description.

A multimodal approach increases the safety, efficacy, and efficiency of treatment, and further research on neurophysiological and molecular markers of response may allow full implementation of a personalized treatment strategy for TRD in both MDD and BD,^114^ (Figure 2).

### Pilot clinical questionnaire for IV ketamine response (TRD in MDD and BD)

**Figure 3.**
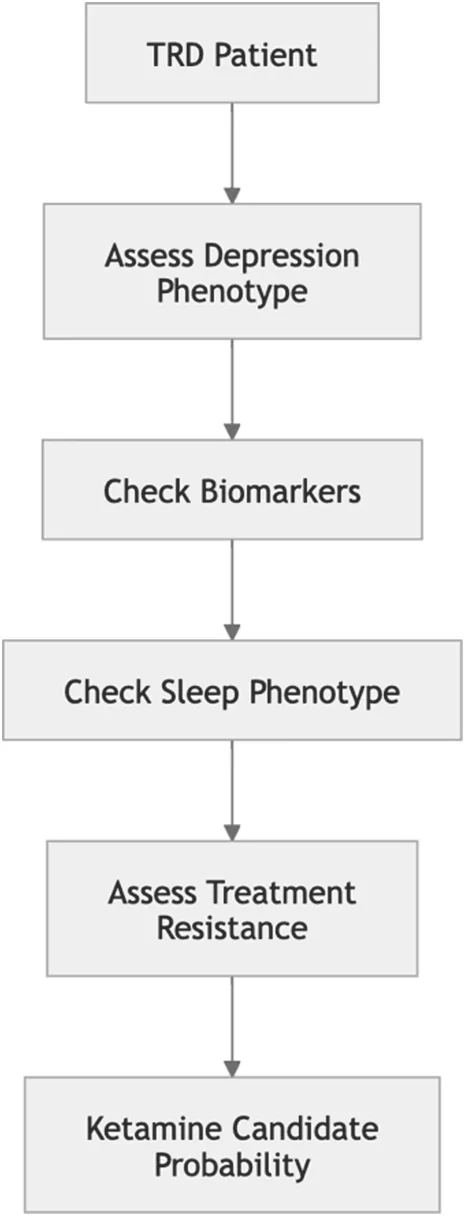
Clinical algorithm for IV ketamine candidate selection.

We developed a pilot clinical questionnaire to integrate predictors of ketamine response; see Supplementary Material for full details (Figure 3). The proposed questionnaire should be regarded as a hypothesis-generating research instrument and has not undergone prospective validation. It is not intended for routine clinical use.

## Limitations and directions for further research

This work has several important limitations that should be considered when interpreting the presented conclusions. First, a significant portion of data on predictors of intravenous ketamine efficacy comes from observational studies, post hoc analyses, and open-label trials. Such a methodological design, while reflecting real-world clinical practice, limits the ability to draw definitive causal inferences and increases the risk of confounding factors.^57^

Another limitation is the heterogeneity of studied populations. Many studies, including patients with TRD analyzed individuals with both unipolar depression and bipolar affective disorder, often without precise differentiation of clinical subtypes, degree of bipolarity, or presence of mixed features. This may lead to underestimation of differences in response to intravenous ketamine between specific diagnostic groups.^22^ ^,^^128^ ^–^^130^

A further limitation is the lack of standardization of treatment protocols. Available studies used varying ketamine doses, different infusion numbers and frequency schedules, and heterogeneous tools for assessing clinical outcomes. This complicates direct comparison of results and formulation of clear recommendations for optimal treatment regimens.^131^ ^,^^132^

Another significant issue is the limited number of long-term studies. Most available data concern short-term efficacy of intravenous ketamine, whereas the durability of therapeutic effect, optimal maintenance strategies, and potential risk of adverse effects with long-term use remain insufficiently studied, particularly in patients with bipolar affective disorder.^133^

Regarding biological factors, it should be emphasized that although reports on the role of neuroplasticity, inflammation, and metabolic markers in ketamine response are increasing, there is still a lack of prospective studies integrating clinical data with objective biomarkers.^34^ ^,^^118^ ^,^^134^ This also applies to sleep parameters, which in most studies were assessed using self-report tools without objective polysomnographic verification.^135^ ^–^^138^

Future research directions should include well-designed, randomized controlled trials that clearly distinguish between unipolar and bipolar depression and analyze clinical subgroups, such as patients with atypical features, sleep disturbances, or comorbid anxiety disorders. Further study of potential mood-stabilizing properties of ketamine and its safety in patients with BD also seems particularly important.^139^ ^,^^140^ Future work should also focus on the development and validation of clinical tools enabling early identification of patients with the highest probability of response, including algorithms incorporating early symptomatic improvement after initial infusions. Such an approach could contribute to more rational and personalized use of intravenous ketamine in TRD.^141^ ^,^^142^ ^,^^143^

Most biomarker findings reviewed in this article originate from small observational studies and post hoc analyses. Replication across independent cohorts is limited, effect sizes are frequently modest, and methodological heterogeneity remains substantial. Publication bias may further inflate the apparent predictive value of reported markers. Consequently, the current evidence base should be interpreted cautiously. Most predictors reviewed here are best viewed as candidate biomarkers rather than clinically actionable tools. Importantly, no biological, genetic, neurophysiological, or metabolic marker discussed in this review has yet demonstrated sufficient predictive performance, reproducibility, external validation, or clinical utility to justify routine implementation in ketamine treatment programs. Although multimodal prediction models represent an important direction for future research, no existing model has yet demonstrated sufficient accuracy or cost-effectiveness to support routine clinical use.

### Relationship between IV ketamine and intranasal esketamine

Although this review focused on intravenous ketamine, the increasing clinical use of intranasal esketamine warrants a brief discussion.^120^ Both interventions act on glutamatergic neurotransmission and demonstrate rapid antidepressant effects in patients with TRD. Intravenous ketamine typically leads to symptom improvement within hours of administration, whereas intranasal esketamine shows a more gradual therapeutic effect across repeated doses during the induction phase. In both cases, maintaining clinical benefit generally requires maintenance treatment; however, esketamine is used within more standardized regulatory frameworks.

Current data suggest that both treatment modalities may share similar predictors of response, such as early symptom improvement, inflammatory markers, and depression phenotype.^144^ However, there is still a lack of head-to-head studies directly comparing the two methods, so it remains unknown whether non-response to intranasal esketamine is associated with a lower likelihood of response to intravenous ketamine. Moreover, there are currently no validated biomarkers available to guide the selection of the optimal therapy, highlighting the need for direct comparative studies of both interventions and further translational research.^46^

## Safety and tolerability of intravenous ketamine

Intravenous ketamine for TRD is generally safe and well-tolerated when administered under controlled conditions by experienced clinicians, with appropriate patient selection and monitoring.^108^ ^,^^131^ ^,^^145^ Most adverse events are transient and mild to moderate, including dissociative and perceptual effects (derealization, depersonalization, visual/auditory disturbances), as well as transient increases in blood pressure and heart rate, nausea, dizziness, headache, and somnolence, typically resolving within hours.^43^ ^,^^136^ ^,^^146^ ^–^^148^ In BD, ketamine may induce hypomanic, manic, or psychotic symptoms if mood stabilizers are inadequate, but risk is low with proper stabilization and monitoring.^108^ ^,^^148^ ^–^^150^ Long-term repeated infusions do not appear to produce cumulative adverse effects, though very long-term safety data remain limited.^146^ ^,^^150^ ^,^^151^ Overall, intravenous ketamine appears to demonstrate a favorable safety profile in both MDD and BD.^152^ ^,^ ^153^ ^,^^154^ ^,^^143^ ^,^^43^ ^,^^155^

## Conclusions

Treatment-resistant depression (TRD) in unipolar depression (MDD) and BD remains a major clinical challenge, highlighting the need for rapid and effective interventions. Intravenous ketamine demonstrates rapid antidepressant, anti-suicidal, and potentially mood-stabilizing effects, making it a promising option in TRD treatment.^34^ ^,^^90^ ^,^^141^

Early symptom improvement after the first or second infusion is a strong predictor of subsequent response.^156^ Patients with atypical depression features, hypersomnia, less advanced treatment resistance, and a positive family history of AUD appear to show the most robust responses.^52^ ^,^^70^ Biological and metabolic factors—including inflammatory biomarkers (monocyte count, AMC), BDNF levels, genetic polymorphisms, vitamin B12, BMI, and childhood trauma—further modulate treatment efficacy. Neurophysiological markers, such as TMS and EEG parameters, provide promising tools for monitoring and predicting response.^89^ ^,^^143^

This review emphasizes practical clinical applications: selecting patients based on depression phenotype, treatment resistance, family and biological factors; monitoring early response to repeated infusions; managing concomitant medications and metabolic parameters; integrating neuroplasticity and neurophysiological biomarkers; and ensuring mood stabilization in BD.^157^ ^,^^158^ ^,^^159^ ^,^^160^ ^,^^128^

Additionally, a pilot clinical questionnaire was developed to integrate predictive factors, facilitating identification of patients with the highest likelihood of response and guiding clinical decision-making.^161^

Limitations include heterogeneity of study populations, variable treatment protocols, and limited long-term data. Future research should focus on randomized, controlled, prospective studies integrating clinical, biomarker, and neurophysiological data, with particular attention to the safety and potential mood-stabilizing effects of ketamine in BD.

Although numerous candidate predictors of ketamine response have been identified, none currently possesses sufficient validation for routine clinical implementation. The field remains largely exploratory, and future large-scale prospective studies are required before biomarker-guided ketamine treatment can become a realistic component of precision psychiatry.

## Supporting information

10.1017/S1092852926101187.sm001 Malewska-Kasprzak et al. supplementary material Malewska-Kasprzak et al. supplementary material

## Data Availability

The data supporting the findings of this study.

## Supplementary material

The supplementary material for this article can be found at http://doi.org/10.1017/S1092852926101187.

## Long descriptions

### Figure 1. Long description

The flowchart is organized into three vertical phases: Identification, Screening, and Included.

1. Identification Phase:

- Records identified from databases: Scopus (n = 372), PubMed (n = 223), E M B A S E (n = 198), and M E D L I N E (n = 137).

- An arrow points right to Records removed before screening: Duplicate records (n = 290).

2. Screening Phase:

- Records screened (n = 639). An arrow points right to Records excluded: Other diagnoses (n = 147) and Non-Human (n = 6).

- Reports sought for retrieval (n = 486). An arrow points right to Reports not retrieved (n = 12).

- Reports assessed for eligibility (n = 474). An arrow points right to Reports excluded: Combined treatment (n = 221), Non intravenous ketamine (n = 59), Case reports (n = 23), Children and adolescents (n = 8), and Other (n = 13).

3. Included Phase:

- Studies included in review (n = 150).

Vertical arrows connect the central boxes from top to bottom, showing the reduction in study count at each stage.

Navigate back to Figure 1..

### Table 1. Long description

The table consists of five columns and three data rows.

Row 1: Inflammatory biomarkers.

- Examples: I L-6, T N F-alpha, A M C.

- Association: Higher A M C is associated with better antidepressant response, while C R P is inconsistent.

- Evidence: Prospective observational studies.

- Clinical readiness: Preliminary evidence.

Row 2: Neuroplasticity biomarkers.

- Examples: B D N F, V E G F, N f L, complement system components.

- Association: Higher baseline or post-infusion B D N F correlates with better response; V E G F and N f L changes reflect synaptic plasticity.

- Evidence: R C Ts and observational studies.

- Clinical readiness: Preliminary evidence.

Row 3: Genetic/Epigenetic biomarkers.

- Examples: B D N F Val 6 6 Met, C Y P 2 B 6, G R I N 2 B.

- Association: Genetic variants influence response and tolerability; D N A methylation and micro R N A expression affect sensitivity.

- Evidence: G W A S and candidate gene studies.

- Clinical readiness: Research use only.

Navigate back to Table 1..

### Table 2. Long description

The table consists of seven rows of predictors:

* Treatment resistance: Features include number of failed antidepressant trials and prior E C T or r T M S. Less advanced resistance correlates with better response, while more advanced resistance correlates with poorer response. Evidence is from observational and clinical studies. Clinical readiness is preliminary evidence.

* Sleep phenotype: Features include hypersomnia, early insomnia, and R E M or S W S changes. Hypersomnia or atypical sleep leads to higher response. Evidence is from clinical studies. Clinical readiness is exploratory evidence.

* Family/Substance history: Features include family history of A U D and personal substance use. Positive family A U D leads to faster and stronger response. Evidence is from clinical studies and meta-analyses. Clinical readiness is exploratory evidence.

* Metabolic factors: Features include Vitamin B 12 levels, B M I, and childhood trauma. Normal or high B 12 and higher B M I lead to better response. Evidence is from observational studies. Clinical readiness is exploratory evidence.

* Neurophysiological/Neuroimaging: Features include f M R I connectivity, E E G or M E G oscillatory patterns, T M S, and M R S glutamate or G A B A ratio. Baseline network disruption and gamma or theta activity are strong predictors. Evidence is from clinical and pilot studies. Clinical readiness is research use only.

* Cognitive/Psychological: Features include working memory, attention, and dissociative experiences. Better baseline cognitive performance leads to more stable response. Evidence is from clinical studies. Clinical readiness is exploratory evidence.

* Concomitant Medications: Features include benzodiazepines and opioid antagonists. High doses may attenuate response. Evidence is from clinical studies. Clinical readiness is preliminary evidence.

Navigate back to Table 2..

### Figure 2. Long description

At the center of the diagram is a black circle with white text reading I V Ketamine Response – T R D. Eight lines radiate outward to rectangular nodes.

* Top-Left: Inflammatory / Immunological. Includes Absolute Monocyte Count (A M C) and C-Reactive Protein (C R P). Subtext: Routine Blood Tests.

* Top-Center: Metabolic. Includes Vitamin B 12 and Body Mass Index (B M I). Subtext: Standard Measurements.

* Top-Right: Clinical Phenotype. Includes Atypical Depression, Moderate Anxiety, and Suicidal Thoughts. Subtext: Clinical Assessment.

* Middle-Right: Treatment Resistance. Includes Failed Antidepressant Trials and Prior E C T / r T M S. Subtext: Medical History.

* Bottom-Right: Early Response Monitoring. Includes greater than or equal to 20% Improvement After 1–2 Infusions. Subtext: Rating Scales.

* Bottom-Center: Family / Substance History. Includes Alcohol Use Disorder (A U D). Subtext: Patient Interview.

* Bottom-Left: Family / Substance History. Includes Benzodiazepines and Opioid Antagonists. Subtext: Current Medications.

* Middle-Left: Concomitant Medications. Includes Benzodiazepines and Opioid Antagonists. Subtext: Current Medications.

Navigate back to Figure 2..
