# Supplementary material for: Multimodal predictors of response to intravenous ketamine in treatment-resistant depression: toward personalized treatment in major depressive and bipolar disorders
Source: CNS Spectr. 2026 Sep 28;31(1):e41. doi: 10.1017/S1092852926101187 (PMC13732598; doi:10.1017/S1092852926101187)
Supplement: Supplementary file 1 [file S1092852926101187sup001.docx]

the Pilot Questionnaire

**Instructions:**

- Completed by the attending physician before starting IV ketamine therapy.
- Some questions allow multiple answers.
- Supports clinical decision-making; does not replace full diagnostics.

#### **I. Basic Data and Diagnosis**

1. **Primary Diagnosis:**
   ☐ Major Depressive Disorder (MDD)
   ☐ Bipolar Disorder (Type I or II)
2. **Current Episode Features:**
   ☐ Depressive with Atypical Features (hypersomnia, increased appetite, “heavy limbs”)

#### **II. Treatment Resistance**

3. **Number of ineffective pharmacotherapy trials:**
   ☐ 2–4
   ☐ ≥5
4. **Previous ECT / rTMS / drug combinations:**
   ☐ Yes
   ☐ No

#### **III. Key Depressive Symptoms**

5. **Dominant symptoms:**
   ☐ Anhedonia
   ☐ Moderate anxiety
   ☐ Suicidal thoughts
6. **Atypical features:**
   ☐ Hypersomnia
   ☐ Increased appetite / weight gain
   ☐ Feeling of “heavy limbs”

#### **IV. Biological / Metabolic Markers**

7. **Vitamin B12:**
   ☐ Normal / high
   ☐ Reduced
8. **Inflammatory markers (AMC / CRP):**
   ☐ Low / moderate
   ☐ Elevated

#### **V. Early Response to Ketamine**

9. **≥20% improvement on depression scale after 1st infusion:**
   ☐ Yes
   ☐ No
10. **Further improvement after 2nd infusion:**
    ☐ Yes
    ☐ No

#### **VI. Final Assessment**

- **Total Score:** … / 16
  **Interpretation:**
- 12–16 → high probability of IV ketamine response
- 7–11 → moderate probability
- 0–6 → low probability; consider alternative strategy
